# Progress in Otology

**Published:** 1900-06-16

**Authors:** 


### Progress in Otology.

(*Continued from page* 172.)

**Ossiculectomy in Chronic Otitis Media.**—R. Lake[1] contributes a paper on this subject with an analysis of 50 cases. He claims that his table of cases shows that the operation is a very successful one, is entirely free from danger to health, and that hearing is often improved by it, and rarely, if ever, diminished. It is not all cases of suppurative otitis which yield to this treatment. Unfortunately many patients require the radical treatment, especially amongst the hospital class. The larger radical operation is required when there are cholesteatomata of the attic, and in cases of destruction of the posterior wall of the osseous meatus. In such cases Lake thinks ossiculectomy should be first performed, and if unsuccessful it should be followed by the larger operation. The operation may be performed for the cure of chronic suppuration, or for the improvement of hearing after discharge has ceased. An uncomplicated case of otorrhœa, which has resisted all forms of treatment for six months, is regarded as being certainly suitable for the operation. If the perforation is situated in the attic or upper and back portion of the drumhead it makes the operation more imperative. Such a perforation more surely indicates a lesion in some part of the ossicular chain; for instance, pus pouring down over the long arm of the incus and head of the stapes first destroys the joint, and then the bones to a variable extent. If these perforations heal up they usually leave marked impairment of hearing power, which loss can often be remedied by a careful operation. A useful practical test of the continuity or discontinuity of the ossicular chain, formulated by A. Cheatle, is to gently stroke the membrana tympani with a Hartmann's probe; in the first instance this is heard and felt, in the second it is felt only. If there are cholesteatomata in the attic, then its outer wall must be removed. This removal of the attic wall is getting to be considered an integral part of the operation, and Lake now does it in all cases. In none of the cases were the ossicles intact; portions of them being destroyed, and more rarely they were firmly ankylosed. The author figures his crotchet-shaped curette which simplifies the removal of the incus, and clears out the attic granulations and detritus. Forty-two of the cases in the list were cured of otorrhœa, and in 21 the hearing power was improved. Transient vertigo occurs in a small proportion of cases. Politzer[2] commented upon the operation of removal of the stapes at the last International Otological Congress. He stated that simple mobilisation of the stapes had only temporary effect on the hearing. Better results were obtained by dividing the adhesions formed between the branches of the stapes and the walls of the niche of the fenestra ovalis. It had been proved that in birds and rabbits, after the extraction of the stapes, a new membrane closing again the fenestra ovalis was formed, and histological examination had shown that no pathological changes were produced in the labyrinth. The operation was no good in sclerosis of the middle ear, because there was then proliferation of the bony tissue of the labyrinth, which continued after the removal of the stapes. In chronic suppurative otitis, the operation had in some cases improved hearing, but in others had destroyed it. Politzer is opposed to removing the stapes in this condition, as it might lead to a spreading of the inflammation into the labyrinth. But when suppuration had ceased, and adhesions remained, he thought there was a distinct future for the operation. E. B. Dench[3] has written on the operation of synechiotomy, *i.e.*, division of adhesions about the stapes. The adhesions are usually between the posterior crus of the ossicle and the corresponding wall of the oval niche, but they may also be found anteriorly and superiorly. In operating cocaine anæsthesia may be used, and the parts sterilised by mopping with perchloride of mercury (1 in 3,000).

The posterior adhesions may have drawn the ossicle out of view, but, knowing its position, the adhesions can be divided without being seen. A sharp knife is introduced through the upper and posterior quadrant of the tympanic ring, and carried inward until the bony wall of the tympanum is encountered. It is then swept downwards, with the point still in contact with the internal tympanic wall. The stapes being thus set free may come into view. After each step in the operation the effect upon the power of hearing should be tested, especially the effect on the lower tone limit. If the above procedure has not improved this, the knife should be passed beneath the crura of the stapes and the adjacent wall of the niche. The author records 26 cases, with improvement in 25. A marked improvement in the power of audition of the opposite ear was frequently noted, subjective noises were often relieved, and commencing inflammation of either the labyrinth or middle ear curtailed.

**Mastoiditis.**—Wessmann[3] is of opinion that the escape of discharge from the inflamed mastoid into the meatus is not so rare as is generally supposed. The condition is merely the extreme issue of the phenomenon known as dropping of the postero-superior wall of the meatus, and is due to the extension of suppuration to the cells which border on the meatus. A small elevation forms in the lower and outer part of the posterior meatal wall, resembling a furuncle, but less tender, moister, softer, and less brilliant. A fistula may form, marked by small granulations, and through this a bent stylet can be passed into a large bony cavity, the antrum, thus differentiating a fistula from mere subperiosteal abscess. The fistula should be enlarged, and drainage provided with a strip of gauze. If this fails the radical operation must be performed. J. F. McCaw[4] also points to swelling of the meatus in the above situation as being indicative of the existence of pus in the mastoid cells. He concludes that in threatened mastoid involvement, and in mild acute cases, conservative treatment should be tried for a week or 10 days, but that operation should be resorted to in acute cases with sagging of the postero superior canal wall, where the infection is of virulent type, and in all cases complicating chronic otorrhœa. Hillard Wood[4] gives the four indications for opening the mastoid cells as laid down by Schwartze, viz.: (1) In acute inflammation of the cells, if the pain, swelling, and fever do not subside after antiphlogosis and free incision; (2) in chronic inflammation of the mastoid, with subacute abscesses or fistulæ; (3) with a sound cortex of the mastoid for cholesteatomata or purulent retention in the middle ear, which cannot otherwise escape, and is associated with dangerous symptoms; and (4) when the mastoid appears healthy, but is the seat of long-continued and unendurable pain, which other means fail to relieve.

[1] Lancet, Mar., 1900. [2] Laryngoscope, Dec., 1899. [3] Jour. Laryng., R. and O., Feb., 1900. [4] Laryngoscope, Mar., 1900.

